# Vertical Hemispherotomy: Contribution of Advanced Three-Dimensional Modeling for Presurgical Planning and Training

**DOI:** 10.3390/jcm12113779

**Published:** 2023-05-31

**Authors:** Alessandro De Benedictis, Alessandra Marasi, Maria Camilla Rossi-Espagnet, Antonio Napolitano, Chiara Parrillo, Donatella Fracassi, Giulia Baldassari, Luca Borro, Antonella Bua, Luca de Palma, Concetta Luisi, Chiara Pepi, Alessandra Savioli, Davide Luglietto, Carlo E. Marras

**Affiliations:** 1 Neurosurgery Unit, Bambino Gesù Children’s Hospital, IRCCS, 4, Piazza S. Onofrio, 00165 Rome, Italy; alessandro.debenedictis@opbg.net (A.D.B.);; 2 Neuroradiology Unit, Bambino Gesù Children’s Hospital, IRCCS, 4, Piazza S. Onofrio, 00165 Rome, Italy; 3 Medical Physics Unit, Bambino Gesù Children’s Hospital, IRCCS, 4, Piazza S. Onofrio, 00165 Rome, Italy; 4 Multimodal Imaging Unit, Bambino Gesù Children’s Hospital, IRCCS, 4, Piazza S. Onofrio, 00165 Rome, Italy; 5 Clinical and Experimental Neurology, Bambino Gesù Children’s Hospital, IRCCS, 4, Piazza S. Onofrio, 00165 Rome, Italy; 6 Intensive Care Unit, Bambino Gesù Children’s Hospital, IRCCS, 4, Piazza S. Onofrio, 00165 Rome, Italy

**Keywords:** epilepsy surgery, vertical hemispherotomy, three-dimensional modeling, augmented reality

## Abstract

Vertical hemispherotomy is an effective treatment for many drug-resistant encephalopathies with unilateral involvement. One of the main factors influencing positive surgical results and long-term seizure freedom is the quality of disconnection. For this reason, perfect anatomical awareness is mandatory during each step of the procedure. Although previous groups attempted to reproduce the surgical anatomy through schematic representations, cadaveric dissections, and intraoperative photographs and videos, a comprehensive understanding of the approach may still be difficult, especially for less experienced neurosurgeons. In this work, we reported the application of advanced technology for three-dimensional (3D) modeling and visualization of the main neurova-scular structures during vertical hemispherotomy procedures. In the first part of the study, we built a detailed 3D model of the main structures and landmarks involved during each disconnection phase. In the second part, we discussed the adjunctive value of augmented reality systems for the management of the most challenging etiologies, such as hemimegalencephaly and post-ischemic encephalopathy. We demonstrated the contribution of advanced 3D modeling and visualization to enhance the quality of anatomical representation and interaction between the operator and model according to a surgical perspective, optimizing the quality of presurgical planning, intraoperative orientation, and educational training.

## 1. Introduction

Hemispheric disconnection has been demonstrated to be an effective treatment in many drug-resistant encephalopathies with unilateral involvement [1,2,3,4]. Starting with the removal of the whole hemisphere (hemispherectomy), subsequent techniques have been proposed, ai-ming to progressively reduce the rate of brain tissue removal in favor of a more conservative strategy based on the isolation of the affected hemisphere from the rest of the brain (hemispherotomy) [5,6,7,8,9].

The three main disconnection approaches are the lateral peri-insular hemisphero-tomy, introduced by Villemure in 1995; the trans-sylvian approach, described by Schramm in 2001; and the vertical parasagittal hemispherotomy, proposed by Delalande in 2007 [10,11,12]. The peri-insular hemispherotomy includes three stages called the supra-insular window, infra-insular window, and insula resection [12], respectively. The trans-sylvian hemispherotomy represents the main variation of the peri-insular approach, consisting of a trans-sylvian resection of the mesial temporal structures through the temporal horn, followed by disconnection of the frontobasal white matter (WM), transventri-cular callosotomy, and occipito-parietal disconnection [11]. The vertical hemispherotomy consists of the transcortical approach to the lateral ventricle, the posterior callosotomy, the fimbria-fornix incision, the latero-thalamic vertical incision, the anterior callosotomy, the frontobasal disconnection, and the trans-caudate lateral incision to the anterior temporal horn [10].

Beyond the peculiar aspects of each technique, the main goal is the interruption of the essential WM structures forming the projection, association, and commissural intra-hemispheric and inter-hemispheric connection systems [13,14,15]. Moreover, as demonstrated by a recent meta-analysis, the lateral and vertical variants provide similar results in terms of both impact on epilepsy outcome and surgical risks [1,4].

One of the main factors influencing optimal epilepsy and neurocognitive outcome in the long term is the completeness of disconnection [16,17]. To this end, a careful awareness of anatomical landmarks and the availability of performant intraoperative neuronavigation systems are crucial in getting an optimal orientation during each step of disconnection. Previous works aimed to describe the surgical technique of vertical hemispherotomy by using schematic representations, pictures from cadaveric dissection, and intraoperative photographs and videos [10,18,19]. Nonetheless, despite the effort to provide an anato-mical representation as realistic as possible, a comprehensive understanding may still be difficult to properly figure out from these studies, especially for less experienced surgeons.

On the other side, neuronavigation accuracy may often be reduced intraoperatively due to brain shift or deformation of the cerebral parenchyma, potentially compromising the completeness of disconnection. Moreover, some etiologies are more frequently cha-racterized by a consistent distortion of brain anatomy, making the recognition of crucial structures and midline preservation more difficult [20,21].

For these reasons, advanced software for three-dimensional (3D) rendering and virtual and augmented reality simulators may constitute valuable tools to enhance the qua-lity of both presurgical planning and intraoperative performance. Although there is a growing implementation of systems for advanced neuroimaging rendering in different domains of neurosurgery requiring a high level of safety and accuracy, such as skull-base, endoventricular and infratentorial approaches, vascular neurosurgery, endoscopic endonasal, and stereotactic procedures, the application of such technology to epilepsy surgery is less frequently reported in the current literature, especially in the pediatric context [22,23,24,25,26,27,28,29,30,31].

In this work, we describe the application of 3D modeling and visualization techno-logy to hemispherotomy procedures according to Delalande’s vertical parasagittal technique for the management of complex forms of epilepsy resistant to anti-seizure medications.

## 2. Materials and Methods

### 2.1. Illustrative Cases

We selected three pediatric cases treated at our Institution for pharmaco-resistant e-pilepsy with hemispheric involvement, including a focal cortical dysplasia case, a hemimegalencephaly case, and a post-ischemic case. All patients underwent presurgical eva-luation, including clinical examination, scalp video-EEG monitoring, neuropsychological testing, and MRI assessment. All MRIs were acquired on a 3T scanner (Magnetom Vida, Siemens, Erlangen, Germany) with a presurgical epilepsy protocol including the follo-wing sequences: 3D MPRAGE, 3D FLAIR, axial FLAIR, axial and coronal TSE T2-weighted images, axial SWI, DTI with 64 directions, post-contrast 3D MPRAGE, and axial phase contrast for neuronavigation and vessel reconstruction, respectively. Tractography reconstruction was performed using MRTrix (http://www.mrtrix.org, version 3.0, accessed on 23 January 2023), MRtrix3, and GitHub (n.d.) (https://github.com/MRtrix3) (accessed on 28 June 2022) with the constraint spherical deconvolution method. Regions of interest (ROIs) were manually drawn to reconstruct the corpus callosum (CC), the optic radiation (OR), the cortico-spinal tract (CST), and the inferior fronto-occipital fascicle (IFOF).

### 2.2. 3D Modeling

3D anatomical reconstructions were performed using the segmentation tools of Mi mics Medical v.25.0 (Materialise, Leuven, Belgium). The DICOM sequences for the specific anatomical structures included T1-weighted sagittal 3D magnetization-prepared rapid gradient-echo (MP-RAGE) sequences for the basal ganglia, ventricles, hippocampus, fornix, fimbria, and optical chiasma; contrast-enhanced T1 MP-RAGE sequences for the venous system; T2-weighted 3D Fluid-Attenuated Inversion Recovery (FLAIR) for the brain parenchyma; and Time of Flight (TOF) angiography for Willis circle segmentation.

**Figure 1:**
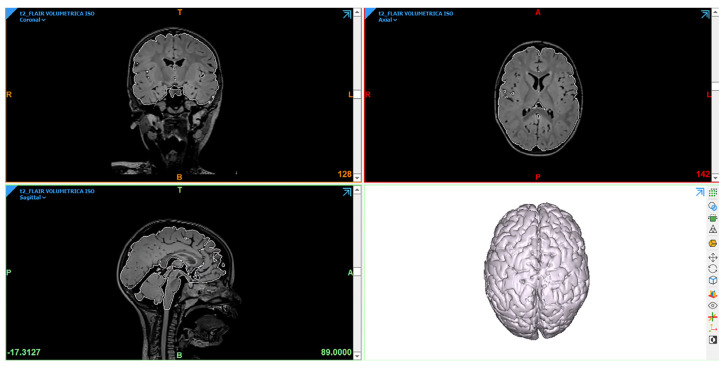
Brain segmentation and 3D rendering using Mimics. The surface contours of the object are overlaid to the 2D MR sequences for a better visualization of the segmentation. (A = anterior, P = posterior, T = top, B = base, L = left, R = right).

After being imported into the Mimics software, the DICOM sequences were first coregistered using the tool “Automatic Segmentation”. After that, different ROIs were created by setting the threshold on the corresponding gray level intensity in the sequence. Different segmentation tools, such as “Multiple Slice Edit”—to edit the mask on the 2D view by marking multiple slices; “Smart Expand”—to dilate the mask within a limited area until an edge is detected; and “Boolean Operations”—to subtract, unite, or intersect two different masks—were applied to make corrections. Each mask was converted into the final stereolithography (STL) object format (Figure 1). The STL files were then imported into the open-source software 3D-Slicer (https://www.slicer.org/) (accessed on 28 June 2022) for visualization of the final models.

### 2.3. Augmented Reality

The AR device selected for our project was HoloLens 2, produced and developed by Microsoft^®^. This is a Head-Mounted Device (HMD), that mounts the Windows 10 operating system and represents an improvement over the first type of HoloLens [32,33,34,35]. All 3D reconstructions were imported into a Unity project. We also associated a set of MRTK scripts to each 3D model, thus allowing user interaction (grab, rotate, enlarge, and shrink) with objects, controlled by hand gestures and their relative inputs via holographic buttons. To set the visibility (i.e., transparency) of the models, a function has been implemented, and a shader has been assigned to the meshes through a script that computes the colors of each rendered pixel. By moving a slider hologram, the user can set the value of the Alpha color parameter of the shader assigned to the meshes. Moreover, 3D structures were displayed or hidden by specific functions based on changing a Boolean variable when the user pressed the corresponding button.

### 2.4. Surgical Procedure

Hemispherotomy was performed according to the vertical parasagittal technique developed by Delalande et al. [10]. Briefly, the patient is placed supine with the head in a neutral, slightly flexed position. A linear transverse incision is performed, allowing for a small parasagittal frontoparietal craniotomy (3 × 5 cm, 1–2 cm from the midline, 1/3 anterior, and 2/3 posterior to the coronal suture). A limited cortical rese-ction (3 × 2 cm) is performed through a small parasagittal frontoparietal craniotomy to reach the central part of the lateral ventricle. After identification of the corpus callosum by following the roof of the lateral ventricle medially, the first step of the hemispherotomy consists of the posterior callosotomy until the splenium by using the ultrasound aspirator. At this level, the resection to the midline led to the exposition of the roof of the third ventricle and the arachnoid over the ambient cistern.

From here, the dissection is pursued laterally to the choroidal fissure behind the pulvinar, disconnecting the posterior column of the fornix at the level of the ventricular trigone. From this point, lateral to the thalamus, a strictly vertical incision is then performed in a posterior-to-anterior direction until the roof of the temporal horn is opened to its most anterior part, interrupting the fibers coming from the insular cortex. Then, the callosotomy is completed anteriorly, considering the medial limit of the pericallosal arteries within the interhemispheric cistern. After a limited resection of the most posterior part of the gyrus rectus, the first segments of the anterior cerebral artery and the optic nerve are visible across the arachnoid. In the last step, all the connections from the anterior temporal lobe, the amygdala, and the frontal lobe are cut through a straight incision oriented laterally through the caudate nucleus from the gyrus rectus to join the anterior temporal horn.

## 3. Results

### 3.1. 3D Modeling

**Figure 2:**
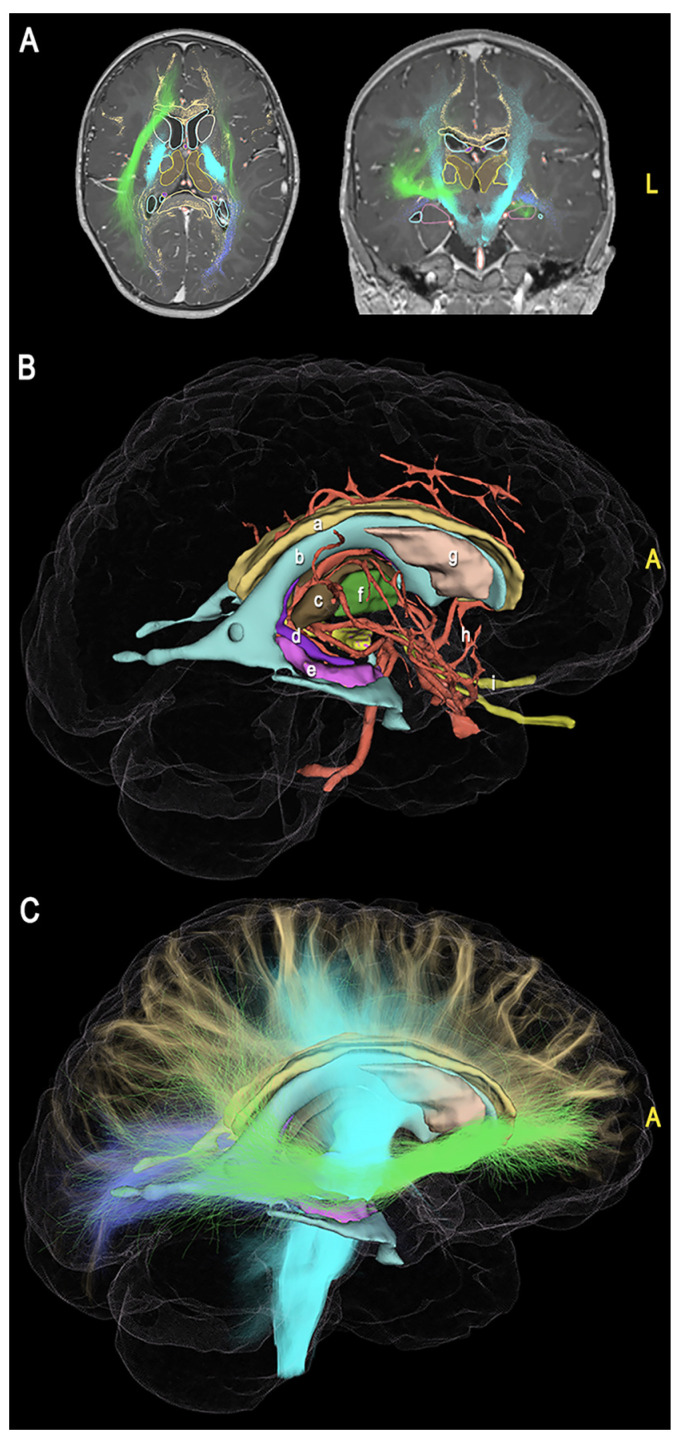
(**A**) Axial (**left**) and coronal (**right**) MR sequences showing the segmented gray and WM structures; (**B**) Comprehensive view of 3D modeling, showing the main brain subcortical structures involved in hemispherotomy procedure, including the corpus callosum (a), ventricles (b), thalamus (c), fornix (d), fimbria and hippocampus (e), putamen (f), caudate (g), main arteries (h), and optic nerves (i); (**C**) Comprehensive 3D representation of the main inter-hemispheric (i.e., the corpus callosum) and intra-hemispheric (i.e., the corona radiata/CST, OR, IFOF) connection systems interrupted during hemispherotomy. (A = anterior, L = left).

For each case, we built a 3D model based on the main neurovascular structures usually involved in vertical hemispherotomy. These included the cortex, venous system (sinuses, main venous efferences), ventricles, thalamus, putamen, hippocampus, fimbria, fornix, head of the caudate nucleus, intracranial carotid artery, pericallosal arteries, and optic nerves. For the FCD illustrative case, we also reconstructed the main WM connection systems, including the corpus callosum (CC), optic radiation (OR), cortico-spinal tract (CST), and inferior fronto-occipital fascicle (IFOF) (Figure 2, Table 1, Video S1).

**Figure 3:**
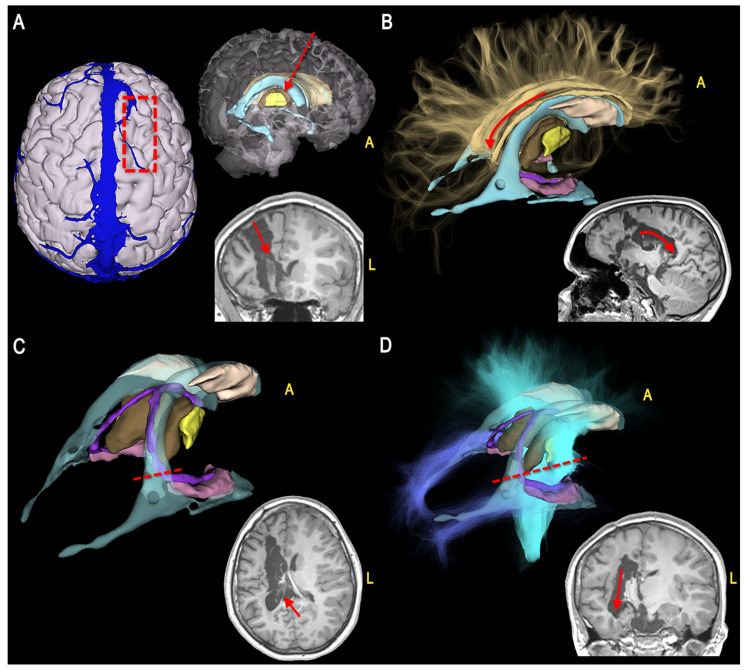
Illustrative Case 1 shows the surgical steps of a right hemispherotomy (first part) in a 2-year-old patient suffering from pharmaco-resistant epilepsy due to multilobar FCD. For each phase, the 3D modeling (**left** side) and the corresponding post-operative MRI (**right** side) are shown. (**A**) Transcortical approach (red dotted rectangle) to the right lateral ventricle (light blue, red arrows); (**B**) Posterior callosotomy (yellow fibers, red arrows); (**C**) Interruption of the fimbria (pink)-fornix (violet) complex (red dotted line, red arrow); (**D**) Unroofing of the vertical (light blue streamlines) and posterior (blue streamlines) connections (red dotted line, red arrow). (A = anterior, L = left).

**Figure 4:**
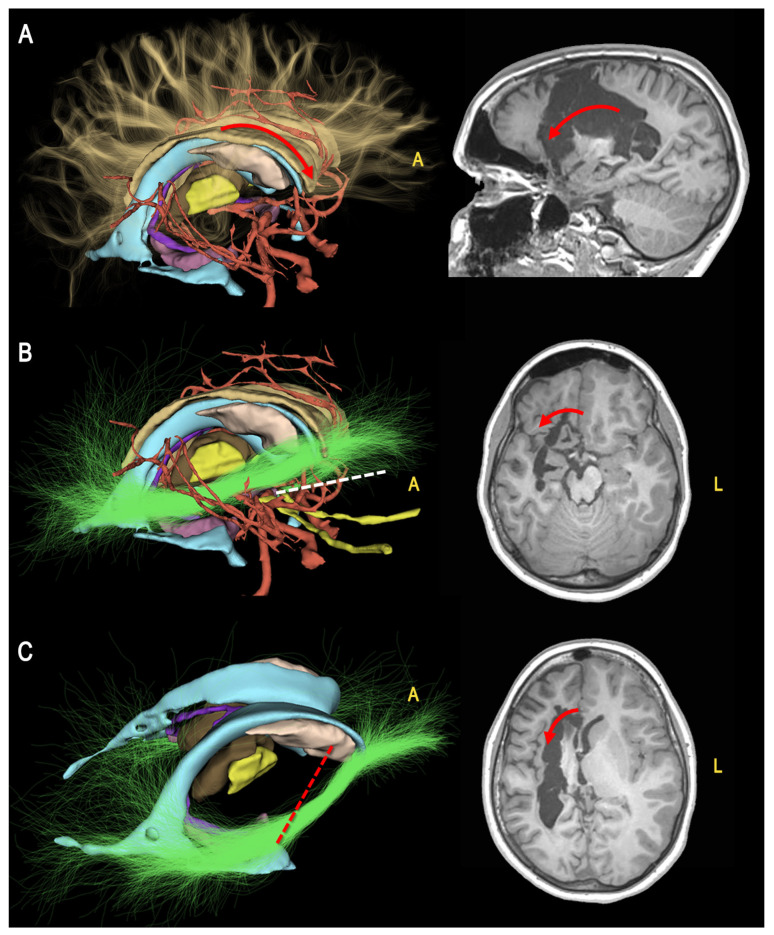
Surgical steps of a right hemispherotomy procedure (second part). (**A**) Anterior callosotomy, having as limit the cistern of the pericallosal arteries (red arrows); (**B**) Frontobasal disconnection having as medial limit the carotid-optic cistern and the intrahemispheric WM connections (e.g., the IFOF) (green streamlines, white dotted line, red arrow); (**C**) The procedure is completed by frontotemporal, medio-to-lateral separation, passing through the head of the caudate nucleus (red dotted line, red arrow). (A = anterior, L = left).

**Figure 5:**
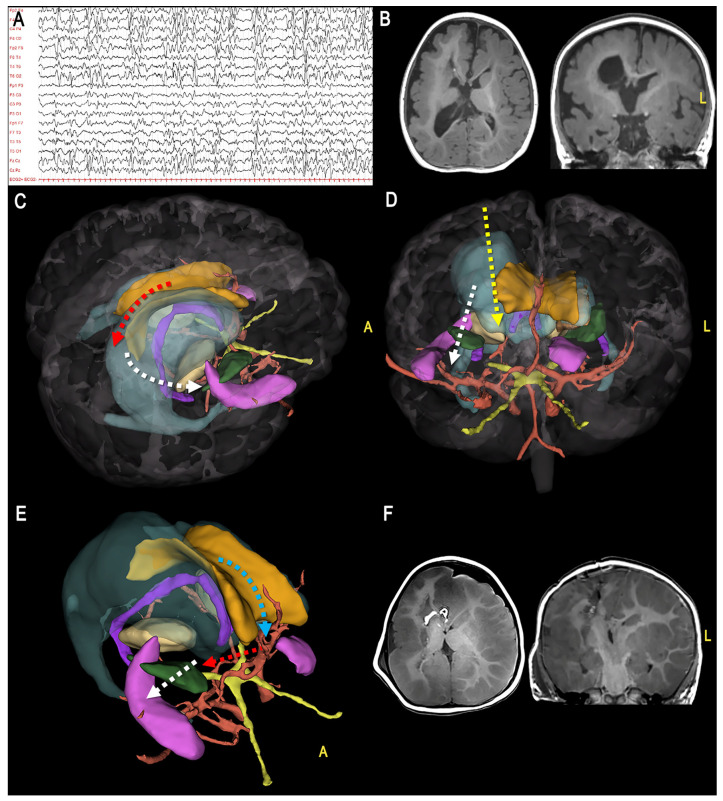
Illustrative Case 2. A 7-month-old girl was admitted to our Institution for recurrent drug-resistant clustered seizures since the age of 1 month, characterized by sudden flexion of the upper limbs and the head, associated with vertical nystagmus; (**A**) The interictal wakefulness EEG showed subcontinous altered activity over the right hemisphere, with multifocal slow and sharp waves intermingled with attenuation phases. Epileptiform abnormalities might be expressed also contralaterally; (**B**) The MRI showed a complex hemimegalencephaly malformation characterized by increased volume of the right hemisphere, diffuse polymicrogyric conformation of the cortex, presence of periventricular heterotopic nodules, alterations of the basal nuclei, and deviation of the right ventricular system; (**C**–**E**) The patient underwent a right vertical hemispherotomy. The 3D model was useful to characterize the 3D configuration of the main target structures during each step of disconnection, including transcortical access to the lateral ventricle (**D**, yellow dotted arrow), posterior callosotomy (**C**, red dotted arrow), fimbria-fornix complex interruption (**C**, white dotted arrow), temporal horn unroofing (**D**, white dotted arrow), anterior callosotomy (**E**, light blue dotted arrow), frontobasal disconnection (E, red dotted arrow), final mediolateral disconnection (**E**, white dotted arrow); (**F**) Postoperative MRI. (A = anterior, L = left)

**Figure 6:**
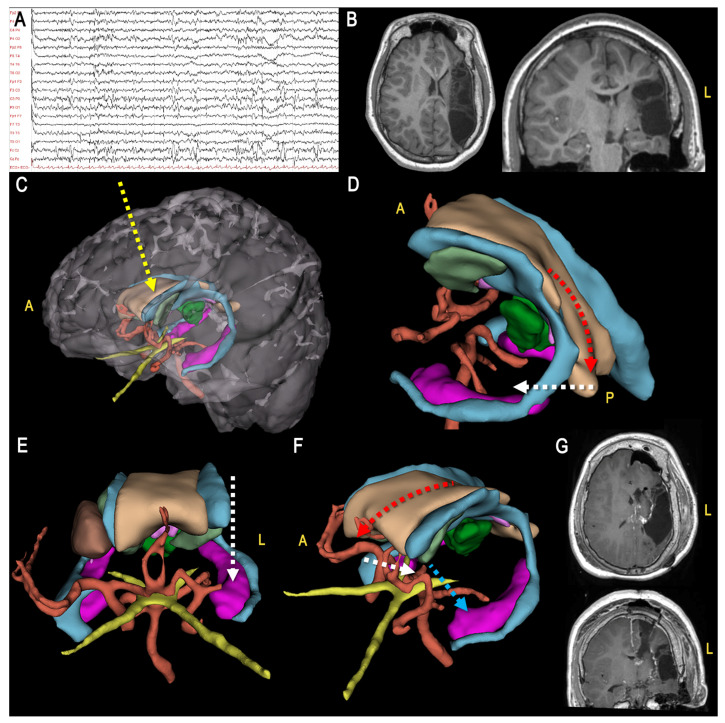
Illustrative case 3. A 14 years-old boy suffered from pharmacoresistant seizures characterized by staring followed by generalized hypertonus. (**A**) Interictal sleep EEG showed recurrent slow waves and spike-and-waves over the left parieto-temporal regions and the vertex; (**B**) The MRI showed a left hemispheric alteration due to post-ischemic perinatal suffering; (**C**–**F**) 3D-modeling showing the main steps of vertical hemispherotomy, including transcortical access to lateral ventricle (**C**, yellow dotted arrow), posterior callosotomy (**D**, red dotted arrow), fimbria-fornix complex interruption (**D**, white dotted arrow), temporal horn unroofing (**E**, white dotted arrow), anterior callosotomy (**F**, red arrow), fronto-basal disconnection (**F**, white dotted arrow), mediolateral (**F**, light blue dotted arrow). (**G**) Postoperative MRI. (A = anterior, L = left).

To highlight the crucial disconnection points, we showed the 3D model of each case oriented according to the progressive surgical steps (Figure 3, Figure 4, Figure 5 and Figure 6).

### 3.2. Augmented Reality

**Figure 7:**
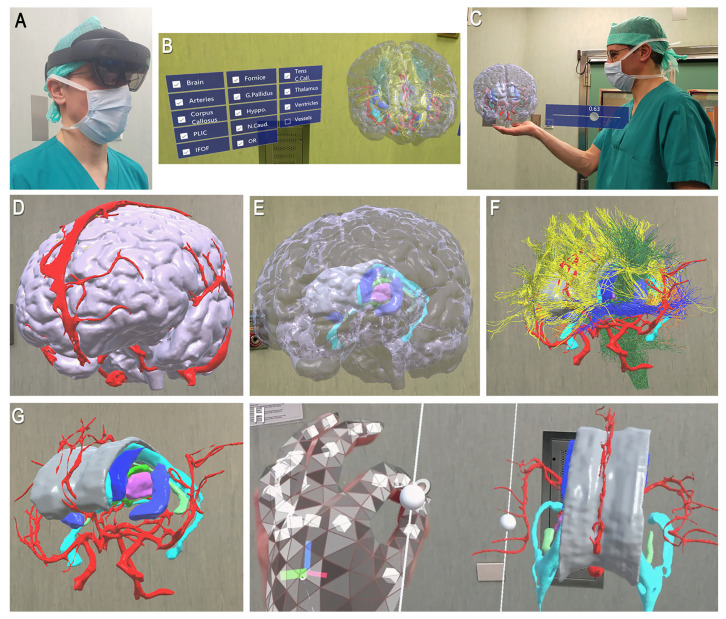
Application of AR technology for virtual representation of the 3D model. (**A**) Operator with glasses; (**B**) The model is visualized on a real background. The menu allows to select different anatomical structures; (**C**) AR technology enables a smart and intuitive interaction between the operator and the model, mainly in two ways. (**D**–**G**) The first option consists of visualization of different anatomical layers, such as the cortex and the superficial vessels (**D**,**E**), the WM pathways (**F**), and the deep structures, including the corpus callosum, the ventricles, the central core, and the Willis polygon (**G**); (**H**) The model can be actively moved by the operator according, for example, to the surgical perspective, to improve the anatomical awareness of the crucial phases of disconnection.

The 3D models were imported into the Hololens system, using the operating room as a real background (Figure 7). We evaluated the different anatomical components selected from the displayed menu. After we showed the direct interaction between the operator and the model, with possible orientations according to different perspectives (Video S2).

## 4. Discussion

The application of advanced 3D modeling and augmented reality to epilepsy surgery has been rarely reported by previous groups, especially in the pediatric literature. Most cases refer to stereo-EEG procedures, in which 3D rendering technology is useful to visualize the exact depth location of electrodes, evaluate their correlation with EEG anomalies, plan the definitive surgical procedure, and facilitate communication with patients and families [36,37].

Concerning open approaches, Wang et al., in 2011, proposed to integrate a direct photographic view of the surgical field with the 3D patient model in the same neuronavigation system to enhance and augment visualization during epilepsy surgery procedures under the image and functional guidance [38]. The following authors described the combined use of 3D simulation models, cadaveric specimens, and intraoperative photographs to clarify the “step-by-step” anatomy of posterior disconnection procedures [39].

In the present study, we evaluated, for the first time to our knowledge, the role of advanced systems of 3D rendering and anatomical visualization for the management of pediatric cases suffering from symptomatic pharmaco-resistant epilepsy forms through the vertical hemispherotomy approach. After its original technical description in a series of 80 patients by Delalande, vertical hemispherotomy has been adopted by growing groups, with different variants proposed by the following authors [10,40,41]. Despite the peculiar aspects discussed in these studies, the main advantages reported for the parasagittal approach with respect to the peri-insular approach include lower invasiveness through a smaller craniotomy, a lower volume of the resected cerebral parenchyma, a decreased risk of injuring the parasagittal draining veins and the healthy hemisphere, and a lower rate of blood loss [18,19].

On the other side, although this approach allows performing the whole procedure from inside an already existing anatomical space, i.e., the ventricular system, an accurate anatomical orientation could be relatively difficult and require a long learning curve, especially for less-experienced groups.

In fact, one of the most important recommendations during vertical hemispherotomy is to follow a systematic sequential order during the different phases of disconnection and to proceed to the next step only when the previous stage has been completed. For this reason, to improve the general anatomical understanding, in the first part of the study, we built a detailed 3D reconstruction of the main WM and gray matter structures to be involved during each disconnection phase and the respective neuro-vascular landmarks (Figure 1, Table 1).

The aim of hemispherotomy is to isolate the pathological hemisphere by interrupting the inter-hemispheric and intra-hemispheric connections while preserving the central core. The central core is a well-defined region between the brainstem and the cerebral lobes. It is composed, from lateral to medial, of the insular surface, extreme capsule, claustrum, external capsule, putamen, globus pallidus, internal capsule, caudate nucleus, stria terminalis, septal region, and thalamus [42]. The superior, posterior, and latero-inferior borders of the central core are encircled by the lateral ventricles and the limbic system (the fornix, parahippocampal gyrus, hippocampus, and amygdala), describing a C-shaped morphology.

The interhemispheric connectivity is constituted by the anterior and posterior commissures and callosal fibers. The main intra-hemispheric WM pathways include the claustro-cortical fibers, interlobar association fascicles (UF, IFOF), forming the external capsule, projection fibers, and OR, forming the IC. The main vascular structures include the internal carotid artery, anterior and middle cerebral arteries, pericallosal arteries, and precentral vein [42,43,44,45,46].

The resulting model can be imported into free access platforms that allow free orientation of the model, selection of specific structures, and simulation of the surgical visual in an interactive way, so improving the anatomical understanding (Figure 3 and Figure 4).

In the second part of the study, we evaluated the adjunctive contribution of 3D technology for the surgical management of the most challenging etiologies. We considered a hemimegalencephaly case and post-ischemic parenchymal damage (Figure 5 and Figure 6). These diseases are frequently characterized by a distortion of the usual anatomical landmarks, including midline deviation, alteration of expected vessel course, and narrower corridors, that can compromise the quality of disconnection towards both incomplete disconnection or damage to the normal hemisphere with consequent worsening impact on epilepsy and neurological outcomes [20,21,47,48].

As shown in the case examples, 3D modeling allows to appreciate the anatomy of the specific case carefully and to translate 2-dimensional to 3-dimensional imagery mentally, helping the neurosurgeon to keep the optimal anatomical orientation while also consi-dering the possible loss of accuracy of neuronavigation systems, which frequently occurs during these procedures.

The 3D models were included in an AR system, allowing a direct simulation of the procedure (Figure 7, Video S2). Virtual and augmented reality technologies are gaining increasing interest as useful educational methods and surgical rehearsal platforms to learn and plan complex approaches requiring a fine-tuned understanding of the spatial relationship between eloquent tissue and a surgical target [22,26,28,29,30,49,50].

This is the first time, to our knowledge, that AR has been applied in the context of the vertical hemispherotomy procedure. AR is a relatively recent technology in which virtual elements (objects, videos, texts) are superimposed in a real-world context [26]. Once the 3D model has been imported, the AR system allows for high-quality visualization of the structures, enabling the operator to select the different anatomical components, orient and observe the model at 360° or according to a surgical perspective (Figure 7). We showed the adjunctive value of AR in reproducing a real-time and immersive direct interaction between the operator and the model during the different phases of the procedure. As reported by previous authors on other applications, AR might improve the accuracy of presurgical planning and the spatial reasoning abilities during a training session of less experienced surgeons or educational programs on epilepsy surgery [22,28,49,50,51,52].

Finally, beyond the well-accepted role of advanced 3D virtual models in presurgical planning, an emerging possibility is to incorporate these tools, especially AR technology, into the surgical theater equipment, that is in the neuronavigation system and even directly into the surgical microscope. Currently, the most frequently reported applications concern spinal surgery and trans-sphenoidal, skull-base, and stereotactic approaches for the treatment of tumors and vascular diseases, with growing implementation across different countries and purposes [24,52].

It is worth noting that the quality of results is still conditioned by not negligible technical limitations, including system delay, inaccuracy of calibration and alignment, optical distortion, intraoperative loss of accuracy associated with the neuronavigation precision due, for example, to brain shift and deformation of target anatomy frequently seen during surgery, duration of the battery, encumbrance, discomfort for the surgeons, and financial accessibility. In this regard, further research is needed to overpass these limits through both further specific advancement of AR technology and by strengthening the strategies already adopted for neuronavigation, such as integration of multiple preoperative and intraoperative imaging techniques to correct brain misalignment [53,54].

Nonetheless, as confirmed by recent studies, AR-based neuronavigation has potential advantages over traditional neuronavigation methods since it provides a real-time 3D visualization of the structures of interest directly in the surgical field, improving the surgical orientation, especially in cases with anatomic variants and in reoperations [24,50,51,52,55,56,57,58,59].

## 5. Conclusions

In this work, we demonstrated the contribution of advanced 3D modeling and visualization to optimizing anatomical awareness for vertical hemispherotomy procedures. For each step of the approach, we provided high-quality pictures showing the main connectivity structures to be interrupted and the crucial neurovascular landmarks to be preserved. Integration of 3D models in an AR system allowed not only the enhancement of the quality of anatomical representation but also the orientation of the models according to a surgical perspective in an immersive and interactive way.

We believe that these tools may be of particular interest during presurgical planning, especially for more complex cases in which the normal anatomy is distorted by the underlying pathology, with a consequently higher risk of intraoperative disorientation and insufficient reliability of neuronavigation guidance. Moreover, such a technology may be helpful to facilitate communication with patients, families, and the epileptology team, as well as for educational purposes in the context of surgical training, by supporting traditional methods. Further development and intraoperative application of 3D-rendering technology by integrating it, for example, with neuronavigation, microscope, exoscope, and aptic feedback technology, will improve the anatomical understanding of the surgical target and adjacent structures.

Extended applications to other approaches for selected forms of epilepsy resistant to anti-seizure medications will contribute to improving the quality of post-surgical outcomes and promote the dissemination of epilepsy surgery culture.

## Figures and Tables

**Table 1:** Summary of the main anatomical landmarks and WM connections during each step of hemispherotomy.

Step	Gray Matter Structures	Vascular Structures/Ventricular Compartments/Nerves	White Matter Pathways
1	Prefrontal cortex, ventricle	Precentral veins Body of the lateral ventricle	/
2	/	Body of the lateral ventricle (posterior part)	Posterior fibers of the corpus callosum
3	Thalamus pulvinar, fimbria/fornix junction	Ambient cistern, internal vein trigone of the ventricle	/
4	Thalamus (medial landmark), insula (lateral landmark)	Temporal horn of the lateral ventricle	Internal capsule (including optic radiation)
5	/	Lateral ventricle (anterior part)	Anterior fibers of the corpus callosum
6	Fronto-basal cortex, head of caudate nucleus	Olfactory nerve, optic nerve, anterior cerebral artery, middle cerebral artery	projecting fibers from the anterior part of the frontal lobe (IFOF, UF)

## Data Availability

The data presented in this study are available on request from the corresponding author. The data are not publicly available due to privacy reason.

## Supplementary Materials

The following supporting information can be downloaded at: https://www.mdpi.com/article/10.3390/jcm12113779/s1, Video S1: 3D animation resuming the main structures and connections involved in vertical hemispherotomy, and the main steps of the procedure; Video S2: 3D representation of the brain model, using the AR system. The operator interacts directly with the model, which is visualized on the real background. The system allows regulation of the transparency of the most superficial layer, to select different anatomical structures, and to orient models in all directions.
